# Next-Generation Liposomal and Nanoparticle-Based Systems for Glioblastoma: Molecular Targeting Strategies to Overcome the Blood–Brain Barrier

**DOI:** 10.3390/ijms27198564

**Published:** 2026-09-25

**Authors:** Joaquin Fiallo Arroyo, Bernardo Castellón, Jose E. Leon-Rojas

**Affiliations:** 1 Escuela de Medicina, Universidad de Las Américas, Quito 170125, Ecuador; joaquin.fiallo@udla.edu.ec (J.F.A.);; 2 Grupo de Investigación Bienestar, Salud y Sociedad, Escuela de Psicología y Educación, Universidad de Las Américas, Quito 170125, Ecuador

**Keywords:** glioblastoma, blood–brain barrier, targeted drug delivery, nanomedicine, receptor-mediated transcytosis, siRNA delivery

## Abstract

Glioblastoma (GBM) remains one of the most lethal and intractable central nervous system malignancies, characterized by aggressive cellular proliferation, profound hypoxia, and an immunosuppressive microenvironment. The primary pharmacokinetic obstacle for systemic interventions is the blood–brain barrier (BBB), which restricts the infiltration of free therapeutic agents. To overcome this limitation, translational nanomedicine has driven the design of next-generation delivery systems, including liposomal vectors and synthetic organic, inorganic, and hybrid nanoparticles. This study establishes an exhaustive analysis of molecular targeting strategies designed to actively hijack physiological transport pathways via receptor-mediated transcytosis (RMT) targeting transferrin, lactoferrin, and LRP1 receptors and adsorption-mediated transcytosis (AMT) using polycationic biomaterials. Terminal selectivity via tumor-specific antigens, such as EGFRvIII and nucleolin (targeted by the AS1411 aptamer), is also discussed alongside smart stimuli-responsive platforms that exploit the tumor microenvironment’s acidic pH, hypoxia, and matrix metalloproteinase activity for localized drug release. Concurrently, the deployment of these nanoplatforms for the intracellular vectorization of advanced genetic material, including siRNA (targeting VEGF and MGMT) and CRISPR/Cas9 complexes, demonstrates significant efficacy in reversing genomic chemoresistance and collapsing tumor neoangiogenesis. Finally, the review addresses critical bioengineering and clinical bottlenecks such as manufacturing scale-up, human BBB complexity, opsonization, and the long-term toxicity of inorganic nanoparticles that currently limit the regulatory transition of these highly promising neuro-oncological nanotechnologies from bench to bedside.

## 1. Introduction

Glioblastoma (GBM) represents one of the most lethal and intractable oncological phenotypes, characterized by aberrant cellular proliferation, profound intratumoral hypoxia, and an aggressive invasiveness orchestrated by a highly immunosuppressive microenvironment and chaotic vascularization. The primary pharmacokinetic limitation for any systemic intervention lies in the strict architecture of the blood–brain barrier (BBB), an intricate anatomical and functional network that relentlessly restricts the passage of the vast majority of free therapeutic agents into the parenchyma [1]. To overcome this physiological obstacle, translational nanomedicine has driven the rational design of next-generation delivery systems, predominantly deploying liposomal vectors and synthetic nanoparticulate platforms. These architectures, whose technical diameter ranges from 1 to 100 nanometers for polymeric or inorganic nanoparticles, and up to 1 micrometer in the case of classic liposomal formulations, provide a critical structural shield against premature enzymatic degradation in the blood, favorably altering the volume of distribution and reducing renal and hepatic clearance [2]. This study aims to establish an exhaustive analysis of the molecular targeting strategies designed to actively hijack the physiological transport pathways of the brain. The operational mechanisms of receptor-mediated transcytosis (RMT) and adsorption-mediated transcytosis (AMT) are examined, alongside the terminal selectivity provided by specific tumor antigens. Concurrently, the study dissects the deployment of these nanoplatforms for the intracellular vectorization of advanced genetic material, including RNA interference machinery (siRNA) and genomic editing complexes (CRISPR/Cas9), with the therapeutic cornerstone of reversing genomic chemoresistance pathways and collapsing the tumor’s cellular neoangiogenesis.

## 2. Molecular Targets for Brain Tumor Delivery

### 2.1. Receptor-Mediated Transcytosis (RMT)

Receptor-mediated transcytosis is the most significant and extensively studied mechanism for the active transport of macromolecules and nanosystems across the BBB and their subsequent internalization into glioblastoma multiforme cells [3]. Endothelial cells of the BBB and tumor cells overexpress a wide variety of specific transmembrane receptors that can be targeted through the surface functionalization of nanoparticles and liposomes with selective ligands. Among these, the transferrin receptor (TfR) has received significant attention because glioblastoma cells overexpress this receptor at levels that can be up to 100 times higher than those observed in normal human astrocytes, a phenomenon driven by the need for ferric iron to sustain their rapid proliferation and aberrant metabolism [3]. When nanosystems whose surface has been functionalized with the glycoprotein transferrin (Tf) are used, internalization into the endothelium occurs predominantly through a clathrin-mediated endocytosis process [4]. This mechanism is physically constrained by the biological diameter of clathrin-coated pits, which ranges from 100 to 150 nanometers, thereby imposing an architectural limitation on the size of therapeutic nanoparticles [4]. Empirically, this design approach has proven effective for drug delivery; for example, studies have documented that the encapsulation of classic agents such as cisplatin in Tf-modified liposomes increases transport across in vitro models of the BBB by nearly 200%, significantly enhancing the drug’s intracellular accumulation [3]. Despite these results, the clinical utility of endogenous transferrin faces a pharmacokinetic obstacle, as the high physiological concentrations of this circulating protein in blood plasma fiercely compete for the same binding sites on the cell surface, saturating the receptor and drastically reducing the efficiency of nanocarrier transcytosis [3]. To overcome the competitive saturation of the TfR, nanomedicine engineering has shifted toward the use of ligands that interact with allosteric domains of the receptor [3]. Surface functionalization with monoclonal antibodies, such as the OX26 and RI7217 clones, allows for non-competitive binding to plasma transferrin and has been shown to significantly increase the intracerebral accumulation of chemotherapeutic agents in orthotopic models [5]. However, the high molecular weight of full-length antibodies can limit their deep penetration into the tumor parenchyma; this has driven the development of single-chain variable fragments (scFvs), which retain high affinity and specificity while exhibiting superior pharmacokinetic profiles and more rapid tissue penetration [6]. The main limitations reported regarding the use of these techniques are that, in vivo, the high concentration of free endogenous transferrin competes for binding sites. There is also molecular shielding of ligands induced by the plasma protein corona, which reverses the charge to negative and reduces the efficiency of transcytosis by more than 50%, while also hindering lysosomal release [6]. Finally, high antibody conjugation density increases off-target macrophage clearance in the spleen due to TfR expression in red pulp and macrophages [6].

Another receptor discussed in some studies is the lactoferrin receptor (LfR); its mechanism is based on receptor-mediated transcellular transcytosis, which depends on interaction with the glycoprotein lactoferrin (a high-affinity receptor closely related to the iron-binding protein transferrin) [4]. It is highly expressed in the capillary endothelium of the human blood–brain barrier and in neurons of the cerebral parenchyma and olfactory regions [4]. Several delivery systems related to this receptor have been described, primarily PEG-PCL nanoparticles surface-modified with lactoferrin. For example, polymeric nanoparticles (PLGA) functionalized with a dual conjugate of lactoferrin (Lf) and folic acid for the delivery of etoposide and lactoferrin-modified rotigotine nanoparticles (primarily targeted for the nasal or nose-to-brain route) [4]. It has been demonstrated that platforms modified with lactoferrin consistently increase in vivo penetration into brain tissue compared to naked vectors in both olfactory and systemic transport. PLGA-NPs doubly conjugated with Lf and folic acid exhibited maximum permeability rates in in vitro blood–brain barrier models, with severe cytotoxicity observed in U87MG cells [4].

Low-density lipoprotein receptor-related protein 1 (LRP1) has also been mentioned in several studies; its mechanism is based on the activation of invagination in the capillary membrane and internalization via vesicle-mediated transcytosis, a strictly ATP-dependent energetic and metabolic mechanism (verified because cooling the model to 21 °C completely blocks the active transport of macromolecules) [7]. LRP1 is widely upregulated in glioblastoma tumor tissue and in the hyperplastic capillary endothelium and tumor-associated vessels. It is also present at high physiological levels in the healthy endothelium of the blood–brain barrier [8]. Several drug delivery mechanisms related to LRP1 have been described, including LbL (layer-by-layer) nanoparticles (with a polystyrene core and an outer shell) functionalized with the low-density peptide Angiopep-2 (AP2/ANG, derived from the Kunitz domain of aprotinin) [8]. Also, self-assembled polyhedral frameworks of nucleic acids (framework nucleic acids, FNNs) encapsulated with the ANG peptide (FNN-ANG) and loaded with small interfering RNA (siMGMT) for combined therapy [8]. Finally, endogenous melanotransferrin (p97) capable of mediating trafficking via the low-density lipoprotein receptor [8]. It has been reported that LbL-AP2 NPs show significant increases in confirmed LRP1-mediated transcytosis, losing the effect if a competitive neutralizing anti-LRP1 antibody is applied [4]. FNN-ANG nanostructures demonstrated a massive increase in their endothelial endocytosis coefficient (bEnd.3), quantified as a 7.7-fold increase at 240 min compared to 10 min baseline readings. In vivo fluorescence efflux measurement post-intravenous injection showed that FNN-ANGs reach their peak intra-parenchymal brain intensity in <1 min, accumulating emission signals that are more than double those of control NPs, with a drastic secondary decrease in off-target renal/hepatic metabolism [4]. Unlike the transferrin receptor pathway, LRP1 via p97 lacks the obstacle of exogenous saturation due to the minimal normal surrounding plasma presence of melanotransferrin [6].

### 2.2. Adsorptive-Mediated Transcytosis (AMT)

Cationic systems and electrostatic interaction are characterized by having a mechanism of interaction with the negative charge of the endothelium that is based on a physical phenomenon supported by non-specific ionic interactions [9]. Endothelial cellular luminal membranes and mucopolysaccharides in the epithelium and vasculature exhibit high anionic potential due to abundant glycosaminoglycans and negatively charged residues [9]. Nanocarriers with positive surface density (high zeta potential) induce a strong electrostatic attraction and cellular adsorption-dependent internalization followed by endocytic invaginations (adsorptive-mediated endocytosis) [4]. The use of various nanoparticles has also been described, such as chitosan biomaterial-based nanoparticles (CS NPs); layer-by-layer assembled nanoparticles (LbL-NPs) coated on peripheral surfaces of a highly polycationic nature, such as poly-(L-arginine); and cationic liposomes used for gene vectorization of plasmids (wtp53) or delivery of the compound zoledronic acid [3,4,7]. Their use has been described in several studies and a contribution to therapeutics has been reported due to kinetic advantages in the BBB crossing process; these provide robust initial gross internalization rates. The chitosan material confers high mucosal adherence and the parallel functional quality of temporarily disrupting and opening tight cellular junctions, facilitating, in addition to intracellular passage, an adjacent passive paracellular crossing for direct drugs in intranasal administration (olfactory pathway) [4]. Likewise, these same studies have reported limitations of these systems, mainly the ultimate lack of oncological specificity and a high rate of harmful collateral interactions with any anionic plasma membrane along the body’s capillaries, as well as the proven induction of aberrant (off-target) endocytosis and the persistent risks of direct cytotoxicity with in vivo hemolysis at extremely high charge densities. The accelerated retention and macrophage clearance caused by organs of the reticuloendothelial system has led to the need for synthetic hydrophilic shielding (such as PEG-PEGylation) to attenuate the overall external charge, thereby compromising the core mechanics of AMT attraction or requiring sensitive linkers to re-expose the charge [3].

### 2.3. Tumor-Specific Antigens

EGFRvIII has been described in some studies, and its mechanism of action is based on the epidermal growth factor receptor variant III (EGFRvIII), which is the result of an in-frame oncogenic deletion of genomic exons 2 to 7. This anomaly condenses a structurally truncated transmembrane receptor lacking the normal extracellular domain, which generates constitutive kinase signal transduction without requiring exogenous ligand binding [10]. It has high tumor specificity since its expression is null in healthy parenchyma, and it is exclusive to malignant glial cell lineages [11]. Base amplification of the EGFR gene is documented in approximately 40% of glioblastomas, of which about half concurrently present the mutated EGFRvIII variant. However, this scenario presents a strongly limiting intrinsic pattern for the development of effective therapies, due to immense intratumoral heterogeneity and a subclonal expression profile of an intermittent nature in both space and time [11]. Current therapeutic strategies are turning toward the design of advanced cellular immunotherapies and nanoformulated vectors functionalized with specific ligands. In the cellular field, bivalent or bispecific chimeric antigen receptor T cells (CAR-T) stand out, designed for co-targeting through the simultaneous recognition of EGFR/EGFRvIII (or the cryptic epitope 806) alongside IL-13Rα2 [11]. To refine specificity and mitigate off-target toxicity, logical engineered cells with SynNotch receptors based on prime-and-kill circuits have been developed; these platforms operate by registering the hyper-specific receptor EGFRvIII exclusively as a primary stimulus of the transcriptional promoter to achieve strictly local priming [12]. On the other hand, precision nanomedicine complements this approach through modified inorganic and polymeric vectors for targeted drug delivery. This includes magnetic gold and iron composite inorganic nanoparticles covalently coupled to the functional monoclonal antibody cetuximab, as well as PLGA and polyvinyl alcohol (PVA) polymeric vectors functionalized with the anti-EGFR antibody panitumumab [11].

Nucleolin has a mechanism of selectivity as it is a critical cellular chaperone of stabilization and genomic packaging proteins that commonly, in healthy homeostasis, inhabits obscure intracellular compartments of the cellular nucleolus [13]. Pathologically, it undergoes a dramatic transmembrane relocation (shuttling protein export) operating on the cell surface (peripheral plasma membrane). It is overexpressed in anomalous oncological lineages of active uncontrolled hyperproliferation in the macroscopic architecture of GBM cells, and is also upregulated in endothelial cells of paraneoplastic neoangiogenesis capillaries immersed in the microenvironment [14]. Among selective ligands, the AS1411 aptamer stands out, which is a purified single-stranded oligonucleotide rich in cytosine and guanine. This sequence can fold three-dimensionally to form G-quartet assemblies [13]. This structure gives it a high binding affinity for nucleolin. For its biomedical use, it has been integrated into several transport systems at the nanometer scale. The main design strategies include hybrid DNA nanospheres functionally linked to the AS1411 chain, as well as semiconductor metal nanocomposites, such as inorganic polymeric selenium nanoparticles coated with this same aptamer (SeNPs) [13]. Three-dimensional platforms based on modified and reactive polyhedral DNA have also been developed, which are known as nucleolin-responsive functional nanostructure networks (NCL-responsive FNNs or FNN-ANGs) [8]. Functional assays show high specificity with proven cell lysis induction. Through quantitative cytometry and fluorescence microscopy, it has been observed that AS1411-functionalized nanospheres bind extensively to glioblastoma multiforme cell lines (U87 and U251), while showing very low or no binding when interacting with healthy human astrocytes (NHAs) in control plates. This targeting activates an endocytosis process that depends on ATP consumption and actin polymerization of the cytoskeleton [8]. This dependence was experimentally verified through specific blockades: the use of deoxyglucose and sodium azide reduced cellular uptake to 50.1%, inhibition with sucrose decreased it to 36.6%, and the application of dynasore limited it to 52.0%. Furthermore, an in vitro competition assay that saturated the medium with free AS1411 aptamer caused a drastic drop in the intracellular incorporation of the guided drug, which fell from 14.34 to 1.1 µmol/10^8^ [13]. On the other hand, FNN-type nanoplatforms react dynamically to nucleolin stimulation on the surface of the GBM. Contact with the receptor causes steric changes from a “closed” to an “open” conformation, allowing the selective release of specific interfering RNA [13]. This mechanism inhibits the anomalous transcription of the enzyme and reverses primary resistance to standard agents such as temozolomide, achieving excellent in vivo biosafety. At the blood–tumor barrier (BTB), these vectors managed to improve the long-term transmembrane perfusion index. After two hours of experimental evaluation, the system exponentially outperformed the passive diffusion of free drugs by inducing sustained transcytosis. Finally, this molecular strategy causes collateral damage to DNA strands and destabilizes the mRNA of the anti-apoptotic protein Bcl-2, increasing the cell death cascade in tumor tissue [13].

## 3. Liposomal Systems: From Bench to Bedside

A promising targeted system therapy for brain cancer consists of the use of liposomes as nanomaterial drug carriers. Liposomes are spherical vesicles, ranging from 20 nm to 1 μm, and are composed of either a monolayer or multilayer phospholipid bilayer, which creates an aqueous interior and a lipidic exterior, similar to a cell membrane (Figure 1); because of this amphipathic property, they are able to carry both hydrophilic and hydrophobic agents like peptides, proteins, antibodies, and chemotherapeutic formulations [2,15,16]. This encapsulation of drugs protects the agent from being degraded prematurely in circulation; therefore, liposomes have proven to be effective for protecting and improving pharmacological stability, enhancing bioavailability, and increasing therapeutic effects [2,17]. When examining the pharmacokinetics of drugs carried by liposomes there is a reduced clearance, a lower volume of distribution (Vd), and an affinity for necrotic tissue with high permeability [18]. Some other benefits of liposomes include their low toxicity and their use as siRNA carriers when they are cationic [2].

Despite all these benefits, liposome-targeted therapy faces some challenges such as limited stability, susceptibility to oxidation and hydrolysis reactions, and the activation of the complement system, increasing cardiovascular and hematological risks and contributing to a rapid clearance from circulation [17,18]. Another problem is the “leaking” of drugs while liposomes are in circulation. Because of this, cholesterol is embedded in the membrane to stabilize it; cholesterol also maintains flexibility to cope with external conditions and protects phospholipids from oxidation [2,17,18]. In addition, liposomes face two major limitations; firstly, there is reduced effectiveness because of rapid sequestration of the vesicles by the mononuclear phagocyte system (MPS) which causes quick clearance from the bloodstream and accumulation in the liver, spleen, and kidneys [15,17,18]. The second difficulty concerns overcoming the blood–brain barrier (BBB) and its transport mechanisms that limit the passage of most drugs [2,17].

As a response to these limitations, conventional liposomes have been improved by the PEGylation process, which consists of the addition of hydrophilic polyethylene glycol (PEG) chains into the surface of the liposome; this can be done through the incorporation of PEG-conjugated phospholipids into the bilayer or the use of lipids that contain an amino group, epoxy group, or diacylglycerol moieties that attract PEG [19,20]. The addition of PEG chains creates a hydrated cloud or “shield” that isolates the liposome from interaction with other particles and blood components, including the MPS, plasma proteins, and lipoproteins; the chemical nature of glycolipids generates a weak negative charge that makes particles less likely to be detected by the MPS [15,18,19,20]. The PEGylation of liposomes alters their structure by increasing their size, molecular weight (MW), and surface density: a higher MW means the liposome has a greater polymer chain layer, providing greater shielding from protein adsorption and recognition from the MPS. Similarly, a greater surface density caused by various PEG molecules close together showed the same effects [17,19,20]. Because of these properties, PEGylation has proven effective for improving liposomes by granting them “stealth” from the MPS, reducing immune clearance, and therefore decreasing their liver uptake and enhancing retention in endothelial tissues, which is useful in the treatment of tumors because of the enhanced permeability and retention (EPR) effect [16,17].

Liposomes mainly cross the BBB by active transport and non-specific endocytosis, but there are methods to enhance this. The PEG coating around the liposome increases its penetration, and they can be further adapted into targeted liposomes by the conjugation of active targeting ligands (like transferrin, folic acid, peptides, and monoclonal antibodies) to receptors expressed in the BBB, which facilitates transcytosis by receptor-mediated endocytosis [1,2,17,21]. PEG liposomes can also effectively penetrate the brain by the use of focused ultrasound (FUS), since it reversibly opens the blood vessels in the BBB [19]. Finally, a promising method of avoiding the BBB that is currently undergoing preclinical and clinical trials is convection-enhanced delivery (CED), in which catheters connected to pumps are placed directly into the brain parenchyma; the pumps generate a constant positive pressure infusion so the agents enter the tissue by ‘bulk flow’ [22,23].

One of the difficulties in cancer treatment is how to target the cancerous cells while causing the minimum toxicity to the non-malignant tissues. There are two ways of targeting neoplastic cells: passive and active targeting. In the first case, the chemotherapeutic agent accumulates in the tissue due to an enhanced permeability and retention (EPR) effect, dependent on the size of the nanoparticle and the vascular characteristics of the malignant tissue: a leaky, disorganized vasculature and minimum lymphatic drainage [24]. In contrast, active targeting refers to the addition of targeting ligands to the surface of nanocarriers so they can be recognized by either overexpressed tumoral or BBB receptors, activating receptor-mediated endocytosis [25,26].

Peptides can be efficient targeting ligands in cases of glioblastoma, with three specific kinds: tumor-homing peptide (THP), cancer-penetrating peptide (CPP), and tumor-penetrating peptide (TPP) [27]. THPs recognize overexpressed receptors on the surface of tumoral cells to later penetrate the cell by the RMT pathway; there is a large arsenal of peptides to target different tumoral characteristics such as immune modulators, the BBB and BBTB, the extracellular matrix, and angiogenesis [27]. On the other hand, CPPs are useful because of their easy cell entry that is independent of receptors; their capacity to penetrate cell membranes is due to their composition of positively charged amino acids which electrostatically bind to the negatively charged cell membrane [27]. In this way, CPPs are able to penetrate the BBB efficiently and have affinity for tumoral cells because of their highly negative cellular layer; however, CPPs are non-specific peptides and could lead to toxicity in normal brain tissues [27,28]. THPs and CPPs separately cannot overcome the challenges of crossing the BBB and targeting cancerous cells directly; thus, two alternatives are proposed: the use of TPPs that consist of phages that pass through vascular walls and accumulate in specific desired tissues, or the use of dual-functioned liposomes that contain both types of peptides [27,28]. Combination therapy has a major advantage: effective targeting of malignant cells decreases the development of drug resistance, which is a common problem in cancer treatment, especially when the treatment is by a single agent [29].

Another effective liposome modification is its conjugation with antibodies or antibody fragments by either covalent linkage with lipids or by chemical modification to increase its hydrophilicity and therefore, its affinity for the liposome’s bilayer [30]. The binding of the antibody to the surface of the liposome can also be achieved by using PEG as a linker [31]. This modification of the surface of liposomes generates immunoliposomes that have high specificity for target antigens, proving effective in trial treatment of glioblastoma that expresses EGFR, GD2, VEGF, and VEGFR2 [32,33,34,35].

Additionally, liposomes can be modified by the addition of aptamers: short oligonucleotides of single-stranded DNA or RNA sequences that can bind specifically to targets in tumoral cells [30]. Some advantages presented by this type of molecule include their ability to adopt any shape to bind to the target ligand, reduced immunogenicity, high reproducibility, stable conformation, and smaller composition when compared to other ligands, making it easier for them to cross the BBB; additionally, aptamers are resistant to organic solvent, variations in temperature and pH, and denaturation–renaturation cycles [31,36]. The therapeutic use of aptamers is still under trial, but some promising ligands for GBM include Gint4.T, CL4, AS1411, GMT8, and the ATP aptamer [37].

Among other targeting techniques for glioblastoma, there is a focus on transferrin receptors (TfRs) and folate receptors (FRs). Both receptors are overexpressed in various kinds of cancers, including brain tumors; TfRs are found in the BBB and are needed by the tumor because of a high iron demand due to cell proliferation, making transferrin a sensible ligand [21,25,28,30,31].

Finally, liposomes can also be modified to respond to external stimuli from the tumor microenvironment, releasing the drug when they face hypoxia, enzymatic activity, changes in pH and temperature, and redox properties [2]. Tumors present an acidic pH (between 5.7 and 6.8) because of an increased glycolysis rate and production of metabolites like lactic acid and CO_2_; liposomes can be modified to take advantage of this characteristic with acid-labile lipids or proteins that decompose the bilayer when faced with an acidic pH, releasing the agent from the liposome [17,30,38]. Similarly, another strategy to target the microenvironment of the tumor is the use of temperature-responsive liposomes that contain thermo-sensitive lipids (like dipalmitoylphosphatidycholine), which are stable at body temperature but destabilize at the hyperthermic site of the tumor (40–42 °C) [17,30,38]. The enzymatic activity of the tumoral environment can help liposomes in two ways: liposomes can either be modified with compounds sensible to highly expressed matrix metalloproteinases (MMPs), or with unfunctionalized prodrugs that are activated by tumoral enzymes [38].

Liposomes are currently under the study of several trials to deliver efficient chemotherapeutic drugs. Doxorubicin (DOX) is a hydrophilic chemotherapeutic agent used for several tumors (including breast cancer, bladder cancer, lymphoma, and Kaposi’s sarcoma, among others) that has been proven effective for glioblastoma cell lines in preclinical trials [39]. DOX affects cancer cells by inhibiting topoisomerase II which represses DNA transcription and disrupts its repair, and by an increase in free radicals that damage cell membranes, DNA, and cellular proteins [39,40]. Some recent studies have synthetized DOX-charged liposomes with different types of targets, like carboplatin, p-hydroxybenzoic acid, and SS31 peptide, obtaining successful results that attack different glioma cells and show less toxicity [39,40,41,42]. One problem that remains is the crossing of the BBB with liposomes that are not modified specifically for this target; however, even with this limitation, there are higher concentrations of doxorubicin in glioblastoma tissue [35]. Some current and completed clinical trials using liposomes to target glioma are included in Table 1.

## 4. Advanced Nanoparticle Platforms

There is a wide variety of nanoparticles being developed for the treatment of glioblastoma. Nanoparticles are small particles that have a size between 1 and 100 nanometers, and because of this characteristic, they have a large surface-to-volume ratio, the ability to penetrate the BBB and small capillaries, and can avoid the MPS [1]. They can be made from organic matter (liposomes, dendrimers, polymers, lipid nanoparticles), inorganic matter (from metal oxides, gold, silver, nanodiamonds, carbon dots, nanotubes), or be a hybrid, and can further be classified into four types of supramolecular systems: polymer-based, lipid-based, biomimetic-based, and inorganic-based [1,26]. These carriers can be further modified by incorporating ligands that serve for active targeting, and PEGylation for “stealth”. The materials used for nanoparticles can either be biodegradable or not; non-biodegradable materials will accumulate in the body, causing toxicity, so biodegradable nanoparticles are preferred as drug delivery systems [43]. Nanoparticles can cause other adverse effects: they can present allergic reactions and induce oxidative stress, DNA damage, and inflammation in healthy cells [1].

Polymer-based nanoparticles can range from 10 nm to 500 nm, and can carry drugs dispersed in their matrix or encapsulated in an inner core; they have been studied as a way to target cancerous cells by interacting with its biomolecules, especially with nucleic acids [26,44]. They offer a wide variety of compositions because of the easy addition of hydroxyl (-OH), carboxyl (-COOH), and amine (-NH2) functional groups; these modifications change their size and charge, targeting different molecules [1,26].

The synthetic polymer poly(lactic-co-glycoid acid) (PLGA) has been widely explored because of its biocompatibility, biodegradation, tunable degradation times, and ability to encapsulate a wide range of molecules including proteins, peptides, bacterial or viral DNA, and various anticancer drugs [43,45]. The molecules of PLGA can be modified by adjusting the ratio of lactic acid (LA) and glycolic acid (GA), which affect properties like encapsulation efficiency, degradation rate, particle stability, and drug release; because of this, PLGA nanoparticles are suitable for a sustained release of anticancer drugs [43,46,47]. Another advantage presented by PLGA is its degradation to lactic acid and glycolic acid, metabolites that enter the Krebs cycle and are further eliminated from the body as carbon dioxide and water, without causing any toxicity [48]. PLGA has been categorized as “generally safe” in FDA-approved drugs, but its use for glioblastoma has not yet been clinically approved; preclinical results have been successful, but clinical trials have been limited [46].

Nanoparticles can also be made out of lipidic molecules, which help transport a great variety of therapeutic agents, including nucleic acids; because of their lipid composition, these carriers are similar to cell membranes and have an enhanced uptake by immune and tumoral cells [1,26]. The composition of lipid nanoparticles (LNPs) consists of cationic lipids built in bi- or multilayer micelles; the cationic regions of LNPs can electrostatically bond to the negatively charged miRNA, mRNA, siRNA, pDNA, and antisense oligonucleotides (ASOs) [1,26]. LNPs are also effective for the transportation of hydrophobic drugs, which usually experience poor solubility and absorption; the lipidic surface encapsulates and protects the drug, granting it transportation and protecting it from degradation [47]. However, cationic LNPs pose a cytotoxic risk because they can disrupt the cell membrane, induce cytoplasm vacuolization, and reduce cell activity; for this reason, neutral lipids like cholesterol, phosphatidylcoline, PEG lipids, and structural lipids are added to reduce toxicity and stabilize the structure of the carriers [26].

Solid lipid nanoparticles (SLNs) are a type of LNPs ranging from 50 nm to 100 nm, composed of water, surfactants, and lipids that remain solid at physiological temperatures [1,44]. They are biodegradable, biocompatible, allow a controlled release of drugs, have long-term stability, can avoid the MPS, and can be loaded with both hydrophobic and lipophilic agents [44]. SLNs have high cell penetration and can enter the central nervous system either by paracellular entry through the transient opening of tight junctions of cells of the BBB or by endocytosis [49]. However, SLNs can face a major problem: they present a low efficiency of encapsulation because the crystallization process of certain lipids leaves the carrier without sufficient space for the load of the therapeutic agent [44].

Inorganic nanoparticles are mainly made from metal components such as quantum dots, metals (gold), hafnium oxide, iron oxide, and mesoporous silica, and because of this, they present optical, magnetic, and electronic properties that react to light, magnetism, and radiation [50,51]. This has resulted in different glioblastoma therapies, the most attractive being photothermal therapy (PTT) and photodynamic therapy (PDT); they involve the use of irradiated infrared light to convert light energy to thermal energy, increasing temperature in the cells containing the photothermal therapeutic agent without causing damage to surrounding tissues [52]. Hyperthermia therapy consists of an increase in temperature to about 41–47 °C, which can damage protein folding, induce apoptosis, change the pH, reduce perfusion and oxygenation to the tumoral site, disassemble the cytoskeleton, enlarge tumor pores, and break double-strand DNA, among other effects [53,54]. The therapy is dependent on the particle size, magnetization, Curie temperature (the maximum temperature of a magnetic particle), and external parameters like the alternating magnetic field and the dissipation of heat from the tumor site [55]. Hyperthermia can also be applied to enhance drug delivery by using metallic nanoparticles with an iron oxide core and a polymer shell that is heat-sensible; when exposed to high temperatures the iron oxide core transfers heat to the shell, which contracts and releases the encapsulated drug [55]. Because of these reasons, inorganic nanoparticles have been considered a promising therapy system for various types of cancer; however, they have poor solubility and result in toxicity problems (especially heavy metal compositions), limiting their therapeutic use [56].

Among inorganic NPs, the most studied are gold nanoparticles (AuNPs) because of their versatile properties that allow surface modification, change in size and shape, and use in light-based therapies [51]. Gold has a high atomic number, translating to a better absorption of X-rays; this makes the NPs radiosensitizers, boosting radiotherapy by increasing radiation absorption and generating reactive oxygen species [49,51]. AuNPs can absorb infrared light and induce surface plasmon resonance (collective oscillation of electrons stimulated) to convert it into heat; plasmonic photothermal therapy may induce disruption of the peritumoral BBB, and increase drug delivery and cytoreductive ablation [53,55]. The temperature and NP properties need to be well monitored because laser heating with AuNPs may result in tumor necrosis and apoptosis, or contrarily, in accelerated tumor growth [53]. Gold NPs have been tested in many preclinical trials: their PEGylation resulted in higher double-strand DNA breaks and expression of pro-inflammatory cytokines in GBM cells—doxorubicin-conjugated AuNPs showed effective penetration of the BBB and accumulation in organoids, gallate-conjugated NPs showed high anti-tumor activity and radiosensitizing effects against U251 GBM cells, and cisplatin-conjugated AuNPs decreased tumor development better than cisplatin alone [1,56]. AuNPs have also shown high affinity for tumor microvasculature, which proved effective in imaging by improving CT contrast and a better visualization of tumoral margins [51].

Another important type of inorganic NP is magnetic iron oxide nanoparticles (IONPs), composed of magnetite (Fe_3_O_4_) and maghemite (γ-Fe_2_O_3_) [50]. IONPs have a specific magnetic domain structure, so they only show magnetic properties in the presence of an external field, which prevents agglomeration in the bloodstream and reduces the risk of embolization [51]. Similarly to AuNPs, they show theranostic potential: they can be used therapeutically to generate interstitial magnetic hyperthermia for protein denaturation and heat-shock protein expression within the tumor tissue, or they can be used in imaging for real-time monitoring of biodistribution and therapeutic response because they act as potent T2-weighted contrast agents [51]. In Europe, the use of IONPs as GBM-adjuvant treatment with stereotactic radiotherapy was approved in 2012; since then, the therapy has been considered safe and has resulted in an increased survival of 7 months when compared to standard treatments [53].

## 5. Overcoming Resistance: Delivering Genetic Material

### 5.1. RNAi Delivery: siRNA Targeting VEGF

Small interfering RNA (siRNA) produces potent and specific post-transcriptional gene silencing by binding in a targeted manner to vascular endothelial growth factor (VEGF) mRNA. This binding triggers the degradation of the transcript and suppresses the translation and expression of the VEGF protein [57]. As a result of this reduction, the activation of signaling cascades that promote the formation of new blood vessels is prevented, and neo-vascularization in the tumor cell microenvironment is blocked. This limits the supply of nutrients and tumor expansion [57]. Vascular endothelial growth factor (VEGF) is the main driver of tumor angiogenesis and is overexpressed in approximately 90% of glioblastoma (GBM) cases. In this context, direct gene-level silencing using siRNA aggressively suppresses both tumor growth and new blood vessel formation [57].

The scientific literature indicates that approaches with extracellular anti-VEGF monoclonal antibodies, such as bevacizumab, elicit temporary radiographic responses. However, they often activate angiogenic escape pathways that limit their overall survival benefit [57]. On the other hand, intracellular inhibition mediated by VEGF-siRNA provides sustained and functional suppression of GBM growth. This occurs because it directly targets the molecular mechanisms that sustain abnormal blood vessel formation within the tumor microenvironment [57]. For this purpose, lipid nanoparticles (LNPs) loaded with VEGF-siRNA were developed. With the goal of conferring tumor targeting, the surface of preformed LNPs was modified using a modular post-insertion strategy mediated by an Fc-region-binding peptide conjugate (FcBP-lipids) [57]. This non-covalent anchoring system actively orients anti-PD-L1 antibodies outward by specifically interacting with their Fc domain, which preserves the antigen recognition function of the Fab region intact without needing to resort to chemical covalent coupling methods. The selection of the PD-L1 antigen as a highly selective cellular entry route was based on its epidemiological profile, given that it is expressed in 35–62% of glioma patients [57]. Studies analyzing in vitro results reported a dramatic increase in cellular uptake of the siRNA, achieving levels approximately 50-fold higher in PD-L1-positive GL261 glioma cells. This optimization in delivery translated into a greater than 70% suppression of VEGF mRNA, which in turn strongly inhibited cell proliferation in the cultured models [57]. On the other hand, analyzing in vivo techniques, it was reported that in a GL261 subcutaneous tumor murine model, repeated intratumoral administration of anti-PD-L1–FcBP-LNPs reduced tumor volume by 65% and final tumor tissue weight by 63% compared to the group treated with control LNPs [57]. This biologically significant tumor regression was sustained by a correlated suppression of intratumoral VEGF protein levels. Furthermore, the formulations demonstrated a favorable safety profile, as they did not alter the body weight of the specimens or induce systemic toxicity. The limitations of this model were mainly that despite the promising results, the experimental design presents critical restrictions. The subcutaneous model used in vivo lacks the necessary validation regarding the penetration capacity through the blood–brain barrier (BBB), and does not reproduce the complex orthotopic intracranial microenvironment characteristic of glioblastoma. Additionally, the administration regimen was limited to localized intratumoral injections, which restricts its immediate systemic applicability in the generalized clinical setting of highly invasive or infiltrating brain tumors [57].

### 5.2. RNAi Delivery: siRNA Targeting MGMT

The enzyme MGMT acts as a critical DNA repair effector by actively removing lesions induced by alkylating agents such as temozolomide (TMZ), thereby preventing the induction of apoptosis in the tumor cell [8]. Through the use of small interfering RNA (siRNA) sequences specifically designed against the MGMT transcript, the targeted degradation of its mRNA is triggered. This post-transcriptional blockade inhibits gene expression and suppresses the production of the repair protein, neutralizing the intrinsic enzymatic resistance of the tumor and restoring cellular sensitivity to genotoxic damage [8]. The intracellular delivery of siMGMT induces a drastic reduction in mRNA and functional MGMT protein levels. By suppressing the activity of this enzyme, DNA lesion repair is prevented, which, in combination with TMZ, exacerbates genomic damage [8]. This phenomenon significantly increases the expression of critical pro-apoptotic markers—such as caspase-3 and Bax—and effectively reverses the characteristic chemoresistance of glioblastoma cells [8]. To optimize the transport of siMGMT, structured nucleic acid-based nanoparticles (framework nucleic acids, FNNs) were designed, constructed through the self-assembly of a six-helix DNA bundle whose primary function is to protect and compartmentalize the oligonucleotide inside. To overcome the biological restrictions of the central nervous central system, the surface of these nanostructures was modified at its ends with the Angiopep-2 (ANG) peptide, which facilitates transcytosis across the blood–brain barrier (BBB) and cell targeting mediated by the LRP-1 receptor [8].

Additionally, the design incorporated conformational control in molecular switches consisting of nucleolin (NCL)-sensitive aptamers located on the side walls of the nanostructure [8]. This security mechanism ensures that the siMGMT remains protected during systemic circulation and is selectively released only upon interaction with the overexpressed nucleolin on the membrane of cells within the tumor microenvironment [8]. Functional assays demonstrated that the FNN-siMGMT-ANG platform exhibits highly efficient penetration and accumulation kinetics over time in targeted glioblastoma cells. In in vivo models, the synergistic administration of TMZ in combination with the nanoparticle (TMZ + FNN-siMGMT-ANG) induced a drastic suppression of the intratumoral MGMT protein. This molecular blockade translated into a translational clinical benefit, prolonging the survival rate of mice with orthotopic tumors in a statistically significant manner compared to those who received TMZ monotherapy [8].

Finally, toxicological biosafety analyses confirmed that the nanostructure possesses excellent systemic biocompatibility. Its integration did not generate histopathological alterations or organic dysfunctions, maintaining a negligible hemolysis rate in hematological assays even when exposed to high concentrations (400 nM) [8].

### 5.3. CRISPR/Cas9 Nanocarriers

The system was encapsulated by combining the messenger RNA of the nuclease (Cas9 mRNA) with a single guide RNA strand (sgRNA). The use of mRNA was chosen over plasmid DNA vectors for the purpose of limiting prolonged intracellular exposure to the nuclease, thereby minimizing immunogenic risk and preventing the induction of off-target genomic cuts [58]. To optimize its translational profile, the Cas9 mRNA was chemically modified through the incorporation of 5-methoxyuridine, a strategy designed to enhance strand stability against cytoplasmic degradation and evade the host’s innate immune activation. Likewise, the system was complemented with sgRNA sequences endowed with advanced structural modifications, ensuring a specific double-strand break (DSB) at the selected locus. The vehicular delivery of the system was carried out using lipid nanoparticles optimized for CRISPR technology (cLNPs). These systems were developed from a library of ionizable cationic lipids incorporating hydrazine, hydroxylamine, and ethanolamine bonds conjugated with linoleic fatty acid chains. Following screening assays, the so-called “Lipid 8” (L8) was selected because it demonstrated an outstanding packaging profile, reaching an encapsulation efficiency of over 90% [58]. With the purpose of enabling systemic administration and ensuring tissue selectivity, the cLNPs were surface-engineered for antibody-mediated targeting (T-cLNPs). This design consisted of coupling anti-EGFR (epidermal growth factor receptor) antibodies to the lipid periphery using a lipid-based single-chain anchoring platform (ASSET technology), allowing the vector to be directed toward the overexpressed tumor cells [58]. Gene-editing strategies focused primarily on the disruption of the PLK1 gene (Polo-like kinase 1), an essential effector for cell cycle progression and the survival of rapidly dividing tumor cells; its molecular inactivation alters chromosome segregation and directly catalyzes the apoptotic cascade. Additionally, the green fluorescent protein (GFP) gene was used as an initial experimental target during the optimization phases, serving as a reporter control to validate cleavage kinetics and quantify editing efficiency through next-generation sequencing (NGS) [58].

## 6. Tumor Microenvironment (TME) Interaction

Glioblastoma has a complex tumor microenvironment (TME) that allows tumor progression, invasion, and immune evasion, which makes therapy more complicated; it is composed of heterogeneous glioma cells (classical, mesenchymal, proneural); glioma stem cells that help tumor initiation, recurrence, and resistance; tumor-associated macrophages (TAMs) that evade immune reaction by suppression of CD8-T cells; myeloid-derived suppressor cells that impair natural killer (NK) T cells and cytotoxic T lymphocytes; cancer-associated fibroblasts that remodel the extracellular matrix (ECM) and grant the aggressive nature of the tumor; endothelial cells with increased permeability and edema; and the extracellular matrix made of collagen IV, glycoproteins, glycosaminoglycans (including hyaluronic acid), and proteoglycans [59]. Tumors overexpress factors like TAMs, especially M2-like TAMs which express and secrete proteases and immunosuppressive factors to avoid adaptive immune responses; additionally, the malignancy, migration, and adhesion of glioma cells is correlated with the increase in components like hyaluronic acid, brevikan, tenascin-C, fibronectin, and thrombospondin [60,61]. Additionally, the ECM has elevated levels of matrix metalloproteinases (MMPs) and oncofetal proteins while having low levels of aggrecan; this increases the tumor’s modulation, invasion, and angiogenesis [59].

The ECM has posed a significant challenge for the treatment of glioblastoma. The overexpression of hyaluronic acid and collagen creates barriers that protect the tumor, making chemotherapeutic attempts to penetrate the tumor ineffective; additionally, the radiotherapy and chemotherapy attempts are also limited because of tumor hypoxia and metabolic stress caused by a diminished supply of nutrients and oxygen [61]. Another challenging characteristic of glioblastoma is its immunosuppressive TME that avoids immune detection and enables immune escape, limiting the effectiveness of immunotherapy [62]. For this reason, nanotechnology has been a promising therapeutic strategy for glioblastoma treatment; however, it also has its challenges. The major limitation is the inefficient delivery of nanomaterials into solid tumors because of a heterogeneous distribution of nanoparticles, which accumulate in the perivascular area and tumor periphery due to the abnormal TME [63]. The vasculature found in tumors is chaotic and exhibits increased permeability because of the overexpression of proangiogenic factors (VEGFR-2); this generates vessels that cannot maintain constant perfusion of the tumor, and therefore, impedes nanoparticle transportation to it [63].

Nanoparticles can be modified to have a more efficient delivery to the tumor; nonetheless, a promising alternative is to target the TME characteristics to make them more favorable and obtain better results with the use of NPs. ECM-focused nanotechnology can have ECM as the target to modify its components, generate nanoparticles that respond to different ECM conditions, degrade the ECM to facilitate drug penetration or disrupt tumor structure, and prevent ECM degradation to maintain its integrity and limit tumor invasion [59]. Novel approaches are focused on MMPs, and there are many methods under investigation; for example, MMPs can be used as targets for active targeting by the use of MMP-sensitive peptides that release the drug when reaching the MMPs [64]. Additionally, MMPs can be degraded to avoid tumor invasion; this can be achieved with nanoparticles that modulate the metabolic pathways of MMPs (peptide re-MM15, cannabinoids, glycoprotein SMAGP) or directly carry MMP inhibitors [64]. Other TME characteristics that can be exploited to increase nanoparticle uptake are the vasculature and collagen density, which can be achieved by attempts to normalize the tumor blood vessels and collagen deposits with anti-VEGFR and anti-TGFβ1 [63].

A major characteristic of glioma TME is the presence of poorly vascularized, hypoxic niches. Hypoxic tumor zones are generated by chaotic microvascular generation and rapidly proliferating tumor cells; for the cells to survive they need to migrate towards the periphery, resulting in the aggressive invasion of glioblastoma, and they need to deplete their oxygen supply and shift towards anaerobic glycolysis for the production of ATP, known as the Warburg effect [59,65]. This metabolic alteration results in the accumulation of lactate and protons, which generates an acidic intracellular pH as well as an acidification of the TME [59].

This can be exploited as an advantage by the development of modified NPs coated with polymers, lipids, or peptides that respond to pH; when exposed to acidic conditions, materials like liposomes and AuNPs have shown an enhanced drug release, causing greater cell death [59]. Another approach is to incorporate NPs in non-malignant cells with inherent hypoxia-targeting abilities (monocytes, macrophages, or neural stem cells) that can access the hypoxic centers via chemotaxis regardless of the vasculature; in experimentation, this showed a prolonged tumoral retention of NPs in tumoral regions distant from blood vessels [65]. In trials, NPs with derivatives of nitroimidazole have been successful as radiosensitizers of hypoxic cells, enhancing radiotherapy and chemotherapy effects in glioblastoma [65,66].

## 7. Challenges in Clinical Translation

Although nanomedicine appears to be a promising brain cancer therapy option, only a few nanoparticle models have been approved by the FDA and EMA [67]. This is a consequence of the gap between preclinical drug development and clinical evaluation due to the reliance on small cohorts, single-arm designs, and short follow-up, with many early clinical trials failing to produce generalizable benefits [50,67]. There are also concerns regarding the toxicity of NPs and cumulative exposure because of their biodistribution, long-term retention in reticuloendothelial organs, ion release, and oxidative stress [50]. The accumulation of inorganic NPs such as gold and iron oxide compounds in non-target tissues are a concern for chronic toxicity [68]. Additionally, many of the cancer nanomedicine formulations are in clinical trials for solid tumors, but few of them are targeted to glioblastoma [67]. These struggles need to be addressed by future investigation and clinical studies that provide information on pharmacokinetics, tolerability, and therapeutic efficacy in human participants with glioblastoma [6].

The manufacturing and scale-up of nanoparticles is another challenge. Even minor variations in formulation or processing parameters can alter NP performance, and their development requires precise engineering and robust manufacturing techniques [46]. The production of NPs lack established scientific paradigms, which end in altered biodistribution, immune responses, and safety; furthermore, it makes predictions and understanding of biological interactions and potential side effects difficult [46].

Even when preclinical rodent trials prove successful, the clinical transition may prove NPs to be ineffective because of the physiological complexity of the human BBB and tumor microenvironment [68]. In vivo human models present challenges difficult to replicate in preclinical trials such as the restrictive BBB, opsonization and clearance of nanocarriers, and NP behavior in the human brain [6,68].

## 8. Future Directions and Conclusions

Evaluation of nanocarriers shows that receptor-mediated transcytosis is the best-known method for endothelial internalization. However, it is limited by significant thermodynamic constraints. Targeted transport to the transferrin receptor (TfR), mainly through clathrin-coated pits that have a size limit of 100 to 150 nanometers, leads to notable increases in the transport of chemotherapeutics across cells. Still, it suffers from significant inefficiency in live systems due to competition from the high levels of serum transferrin. This issue worsens because of the plasma protein corona that forms around the nanoparticles. This corona changes the surface charge of the nanoparticles and reduces transcytosis rates by more than 50%, while also increasing non-specific clearance in the spleen by macrophages.

Given these challenges, the LRP1 target, accessed using peptides like Angiopep-2, has demonstrated a specific role in ATP-dependent transport across cells. This approach avoids the saturation issues related to iron, leading to much higher accumulation rates of the nanoparticles in tissue compared to similar methods. As for adsorption-mediated transcytosis (AMT), it uses polycationic materials like chitosan. These materials can temporarily disrupt tight junctions and create immediate electrostatic interactions with negatively charged endothelial mucopolysaccharides. However, this electrostatic method has a major drawback: it lacks tissue specificity, which can cause systemic hemolysis and unwanted endocytosis. This necessitates adding hydrophilic shields, like PEGylation, to extend circulation time.

In the tumor microenvironment, the EGFRvIII target shows no expression in healthy brain tissue, but its high variability causes dangerous clonal escape events. This resistance can currently only be overcome by the use of secondary antigens through combined methods such as SynNotch cellular technology. At the cell level, the AS1411 DNA aptamer that targets the nucleolin inside cells relies on actin polymerization for internalization. It selectively kills cancer cells without harming healthy glial cells, gradually overcoming the resistance of the blood–tumor barrier over time.

In genetic applications, encapsulating siMGMT within framework nucleic acids (FNNs) linked to nucleolin significantly reduces tumor DNA repair. This restoration allows alkylating agents like temozolomide to be fully effective again. Meanwhile, lipid formulations linked to anti-PD-L1 Fc domains can silence VEGF mRNA, reducing excessive blood vessel growth by 65% without needing permanent anchors. Additionally, in vivo editing using antibody-targeted lipid nanoparticles (cLNPs) helps knock out essential survival genes like PLK1. This uses modified Cas9 mRNA to avoid immune responses and prevent unwanted changes to the genome.

The shift in neuro-oncological nanomedicine from research labs to clinical use requires careful adjustments to significant engineering and translation challenges that delay regulatory approval from agencies like the FDA or EMA. The step from animal models to human applications shows serious differences in how things work. This is due to the complex anatomy and function of the human blood–brain barrier, as well as the strong opsonization processes of the human immune system. These factors make it hard to rely on preclinical results from short-term studies with single-arm designs.

A major technical challenge concerns scaling up the manufacturing process. Small changes in polymerization, ligand ratios, or the amount of PEGylation can greatly affect the surface characteristics and toxicity of the nanoparticles in live systems. This calls for a level of standardization that the biomedical field has not yet achieved. To reduce long-term toxicity from build-up in the reticuloendothelial system—especially when using dense nanoparticles like gold or iron oxide for heat therapies—it is crucial to switch to inorganic materials that have controlled biodegradation rates or purely organic carriers.

To overcome the immune suppression and barriers created by the low-oxygen extracellular matrix, carriers need to focus on simultaneous or dual-targeting methods. Combining cell-penetrating peptides with tumor-homing peptides on one liposome can help avoid the blood vessels and target the tumor tissue directly. This strategy can minimize the chances of the tumor escaping treatment and protect nearby astrocytic networks.

The future of in vivo gene editing for the central nervous system will need a new level of optimization in the design of lipid nanoparticles to fit larger ribonucleoprotein complexes. Besides the packaging, it will also be important to include specific modulating sequences and proteolytic linkers that activate or release the nuclease when exposed to acidic conditions, low oxygen, or heightened matrix metalloproteinase activity. This approach will ensure that chromosomal cuts remain inactive throughout the body and only occur in the metabolic center of the glioblastoma.

## Figures and Tables

**Figure 1:**
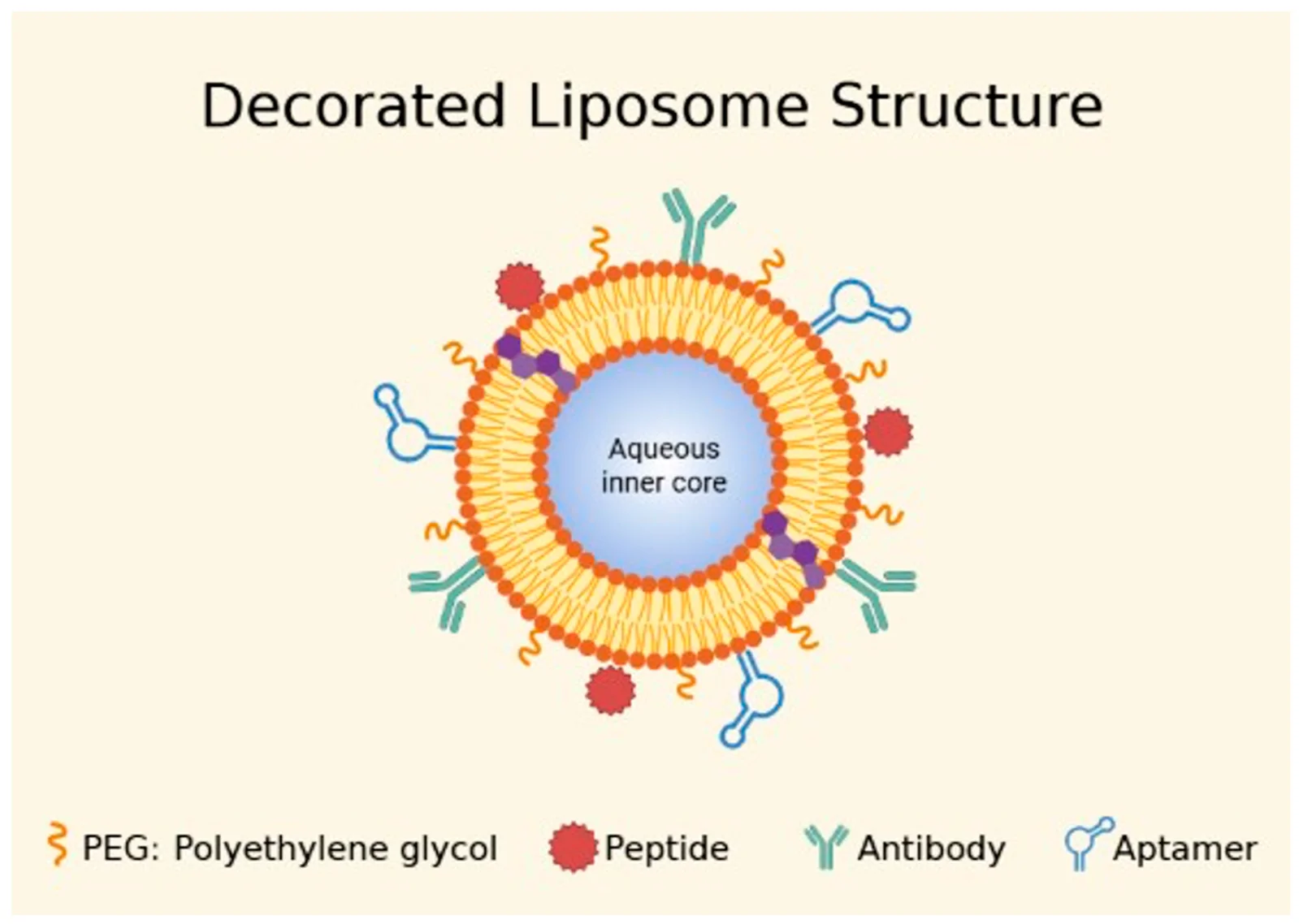
The liposome structure is composed of a monolayer or multilayer phospholipid bilayer, simulating a cell membrane and generating an aqueous pore and a lipidic exterior. Similar to a cell membrane, cholesterol can be embedded in the phospholipid bilayer to stabilize its structure and grant flexibility. Liposomes can be functionalized for active targeting of cells by decorating its surface with peptides, aptamers, or antibodies. Groups of polyethylene glycol (PEG) can also be added to the surface to help liposomes avoid the MPS and penetrate the BBB.

**Table 1:** Clinical trials of liposome nanoparticles that target glioblastoma or glioma registered in ClinicalTrials.gov.

Description of the Liposome	Patient Eligibility Criteria	ClinicalTrials.gov ID	Aim of the Study	Status
186 rhenium nanoliposomes (186RNLs) administered by convection-enhanced delivery (CED)	Recurrent or progressive malignant glioma (grade III/IV) after surgical, radiation, and/or chemotherapy treatment	NCT01906385	Evaluation of multiple separate dose levels (dose escalation)	Recruiting
186RNLs administered by an intraventricular route (via CED)	Adults with stage III/IV glioma following initial treatment with 185RNLs (at least 90 days from prior dose)	NCT05460507	To determine the safety and efficacy/tolerability of 186RNLs in recurrent treatment	Recruiting
Pegylated liposomal doxorubicin (PEG-Dox)	Adults with newly diagnosed glioblastoma	NCT00944801	To determine if the addition of PEG-Dox to temozolomide (TMZ) and radiotherapy improves the survival of patients	Completed
Liposomal curcumin (LC) administered intravenously	Adults with newly diagnosed high-grade malignant gliomas (grade III/IV, including GBM, astrocytoma, gliosarcoma, H3K27M mutant diffuse midline glioma)	NCT05768919	To assess the tolerability, safety, and efficacy of LC in combination with radiotherapy and TMZ	Recruiting
Nanoliposomal CPT-11	Recurrent high-grade malignant gliomas (GBM, gliosarcoma, anaplastic astrocytoma, anaplastic oligodendroglioma, anaplastic mixed oligoastrocytoma)	NCT00734682	To compare survival with CPT-11 with that of historical controls and assess toxicities	Completed
Liposomal doxorubicin	Adults with newly diagnosed pathologically proven glioblastoma, isocitric dehydrogenase-1/2 wild-type.	NCT05864534	To establish whether it is safe and feasible to administer liposomal doxorubicin with balstilimab and botensilimab accompanied by sonication, and to identify whether the treatment is effective in treating glioblastoma	Recruiting
Liposomal verteporfin administered intravenously (IV)	Patients with recurrent or progressive grade IV glioma (glioblastoma) with mutant or amplified EGFR that have received standard first-line therapy.	NCT04590664	To evaluate the safety and tolerability of successively higher doses of verteporfin and determine the maximum tolerated dose in EGFR-positive glioblastoma.	Recruiting
Liposomal transcrocetin (L-TC) administered intravenously (IV)	Histologically confirmed diagnosis of glioblastoma in non-operable patients or elderly patients	NCT06477939	To study the outcome of adding or not adding L-TC with concomitant hypofractionated radiation therapy and temozolomide	Not yet recruiting
RNA-lipid particle (RNA-LP) vaccines including mRNA pp65 and tumor RNA from each patient’s tumor tissue	Adults with histopathologically proven glioblastoma with evidence of progression	NCT06389591	To demonstrate the manufacturing feasibility and safety, and to determine the maximum tolerated dose, of RNA-LP vaccines.	Recruiting
Liposomal doxorubicin (caelyx) administered intra-arterially with carboplatin	Adults that have failed the standard first line of treatment (Stupp protocol) and that have been diagnosed with recurrent multiforme glioblastoma	NCT06356883	To compare the administration of carboplatin combined with caelyx vs. carboplatin combined with etoposide phosphate, by evaluating tumor response using the RANO criteria	Recruiting
RNA-lipid particle (RNA-LP) vaccine: autologous total tumor mRNA and pp65 full length (fl) lysosomal-associated membrane protein (LAMP)-loaded DOTAP liposome vaccine administered intravenously.	Adult patients (≥21) with newly diagnosed de novo MGMT unmethylated glioblastoma (grade IV), and pediatric patients (>3 and ≤25) with newly diagnosed high-grade glioma (grade III or IV)	NCT04573140	To demonstrate the manufacturing feasibility and safety, and to determine the maximum tolerated dose, of RNA-LP vaccines	Recruiting

## Data Availability

No new data were created or analyzed in this study. Data sharing is not applicable to this article.
